# Relationship Between Perceived COVID-19 Risk and Change in Perceived Breast Cancer Risk: Prospective Observational Study

**DOI:** 10.2196/47856

**Published:** 2024-12-02

**Authors:** Ryan Baxter-King, Arash Naeim, Tina Q Huang, Karen Sepucha, Annette Stanton, Aaron Rudkin, Rita Ryu, Leah Sabacan, Lynn Vavreck, Laura Esserman, Allison Stover Fiscalini, Neil S Wenger

**Affiliations:** 1 Department of Political Science UCLA Los Angeles, CA United States; 2 Center for SMART Health, Clinical and Translational Science Institute David Geffen School of Medicine at UCLA Los Angeles, CA United States; 3 UCLA School of Medicine Los Angeles, CA United States; 4 Health Decision Sciences Center Massachusetts General Hospital Harvard Medical School Cambridge, MA United States; 5 Department of Psychology UCLA Los Angeles, CA United States; 6 Department of Psychiatry/Biobehavioral Sciences UCLA Los Angeles, CA United States; 7 Breast Care Center UCSF San Francisco, CA United States; 8 Department of Communication UCLA Los Angles, CA United States; 9 Division of General Internal Medicine and Health Services Research UCLA Los Angeles, CA United States

**Keywords:** breast cancer, COVID-19 risk perception, cancer screening, anxiety, cancer, COVID-19, prevention, medical care, screening, survey

## Abstract

**Background:**

Whether COVID-19 is associated with a change in risk perception about other health conditions is unknown. Because COVID-19 occurred during a breast cancer study, we evaluated the effect of COVID-19 risk perception on women’s breast cancer risk perception.

**Objective:**

This study aims to evaluate the relationship between perceived risk of COVID-19 and change in perceived breast cancer risk. We hypothesized that women who perceived greater COVID-19 risk would evidence increased perceived breast cancer risk and this risk would relate to increased anxiety and missed cancer screening.

**Methods:**

Women aged 40-74 years with no breast cancer history were enrolled in a US breast cancer prevention trial in outpatient settings. They had provided breast cancer risk perception and general anxiety before COVID-19. We performed a prospective observational study of the relationship between the perceived risk of COVID-19 and the change in perceived breast cancer risk compared to before the pandemic. Each woman was surveyed up to 4 times about COVID-19 and breast cancer risk perception, general anxiety, and missed medical care early in COVID-19 (May to December 2020).

**Results:**

Among 13,002 women who completed a survey, compared to before COVID-19, anxiety was higher during COVID-19 (mean T score 53.5 vs 49.7 before COVID-19; difference 3.8, 95% CI 3.6-4.0; *P<*.001) and directly related to perceived COVID-19 risk. In survey wave 1, anxiety increased by 2.3 T score points for women with very low perceived COVID-19 risk and 5.2 points for those with moderately or very high perceived COVID-19 risk. Despite no overall difference in breast cancer risk perception (mean 32.5% vs 32.5% before COVID-19; difference 0.24, 95% CI –0.47 to 0.52; *P*=.93), there was a direct relationship between change in perceived breast cancer risk with COVID-19 risk perception, ranging in survey wave 4 from a 2.4% decrease in breast cancer risk perception for those with very low COVID-19 risk perception to a 3.4% increase for women with moderately to very high COVID-19 risk perception. This was not explained by the change in anxiety or missed cancer screening. After adjustment for age, race, education, and survey wave, compared to women with very low perceived COVID-19 risk, perceived breast cancer risk increased by 1.54% (95% CI 0.75%-2.33%; *P*<.001), 4.28% (95% CI 3.30%-5.25%; *P*<.001), and 3.67% (95% CI 1.94%-5.40%; *P*<.001) for women with moderately low, neither high nor low, and moderately or very high perceived COVID-19 risk, respectively.

**Conclusions:**

Low perceived COVID-19 risk was associated with reduced perceived breast cancer risk, and higher levels of perceived COVID-19 risk were associated with increased perceived breast cancer risk. This natural experiment suggests that a threat such as COVID-19 may have implications beyond the pandemic. Preventive health behaviors related to perceived risk may need attention as COVID-19 becomes endemic.

## Introduction

COVID-19 posed a new serious threat that caused Americans to change how they led their lives. People who perceived themselves to be at higher risk for developing COVID-19 were more likely to engage in protective behaviors [1,2]. Lack of access, higher perceived risk of COVID-19, more symptoms of anxiety or depression, and risk factors for severe COVID-19 were associated with missed medical care and cancer screening [3]. The longer the pandemic persisted, the greater the importance of the influence of COVID-19 on activities to promote health, such as preventive behaviors, including cancer screening, which is associated with earlier detection and better survival [4]. Preventive behaviors, such as mammograms, are dependent—at least in part—on the perceived risk of the condition [5,6]. However, little is known about how the perceived risk of a condition is affected by the imposition of a new risk from another condition. COVID-19 created a natural experiment in the midst of a breast cancer prevention study, permitting elucidation of how a new perceived risk affects the perception of a prior health risk.

Prior to the pandemic, the WISDOM (Women Informed to Screen Depending On Measures of Risk) breast cancer prevention study enrolled women, elicited their perceptions of the risk of breast cancer and levels of anxiety, and presented personalized or routine screening recommendations. During COVID-19, WISDOM added surveys to study the relationship between the perceived risk of COVID-19 and perceived breast cancer risk and explored factors associated with the relationship. Because of evidence that anxiety and distress related to risk perception [7,8] and evidence that people were missing cancer screening due to COVID-19 concerns [3], we hypothesized that individuals who perceived greater COVID-19 risk would have increased perceived breast cancer risk during the contemporaneous survey wave and that this risk would be related to increased general anxiety [9] and missed cancer screening [10].

## Methods

### Study Sample and Baseline Data

In this prospective observational study, women aged 40-74 years with no breast cancer history were enrolled at mammogram facilities and physician offices, and via health system communication and media [11]. At study entry, women provided demographic information, estimated their risk of breast cancer, and answered questions about anxiety. Numerical breast cancer risk was assessed by asking “What do you think your chance is of developing breast cancer in your lifetime? Please choose a number between 0% (no chance of breast cancer) and 100% (definitely will get breast cancer)” [12]. General anxiety was assessed using the 4-item PROMIS (Patient-Reported Outcome Measurement Information System) short form [13]. The raw score was rescaled into a standardized T score, with higher scores indicating more anxiety. A score of 50 (SD 10) represents the mean score for the general population. A change of 2.5 T score points appears to represent a minimally important difference [14]. Surveys were web based. The pre–COVID-19 survey was completed a mean of 9 months before the pandemic began.

### Surveys

Participants were asked to complete 4 supplemental COVID-19 surveys, collected from May 10 to June 15, 2020; from July 11 to August 21, 2020; from October 5 to 28, 2020; and from December 8 to 30, 2020. Approximately 25,000 women were sent the optional web-based survey each wave (Table S1 in Multimedia Appendix 1). Participants received an initial email and follow-up email reminders.

Surveys included the items on breast cancer risk perception and general anxiety that were completed in pre–COVID-19 WISDOM surveys. Perceived COVID-19 risk was assessed with an item adapted from the National Cancer Institute’s Health Information National Trends Survey [15]: “How likely is it that you will get COVID-19 in the next 30 days?” with response options of “Very low,” “Moderately low,” “Neither high nor low,” “Moderately high,” or “Very high.” The survey asked whether the respondent or a household member believed they had COVID-19 and whether the respondent had significant medical diagnoses (heart disease, lung disease, diabetes, hypertension, cancer, and others).

The survey asked about missed medical appointments as follows [3]: “Over the last 2 months, have any of your health care providers canceled or postponed scheduled visits or services for physical or mental health?” Response options were “Yes,” “No,” “I did not have anything scheduled,” and “Not sure.” Respondents also were asked if they had canceled or postponed scheduled visits or services. These 2 items were combined to describe whether the respondent had a medical visit that was canceled or postponed. Concerning cancer screening, the survey asked the following: “Over the last 2 months, have you canceled or postponed getting routine cancer screening (breast cancer mammography, colonoscopy, etc)?” with the response options of “Yes,” “No,” “I did not have anything scheduled,” and “Not sure.” Concerning the future, respondents were asked “In the upcoming 2 months, do you plan to cancel or postpone getting routine cancer screening (breast cancer mammography, colonoscopy, etc)?” and the same question was asked concerning visits or services for physical or mental health.

### Ethical Considerations

This study was approved by the University of California Los Angeles Institutional Review Board (#20-000786) and the University of California San Francisco Institutional Review Board (#15-18234). All participants provided written informed consent. Data were deidentified prior to analysis. No compensation was offered for study participation or survey completion.

### Statistical Analysis

All survey respondents were eligible for inclusion in the analysis, even if they did not complete all 4 surveys or did not have baseline data for anxiety or perceived breast cancer risk. We calculated the change in perceived breast cancer risk by subtracting the pre–COVID-19 survey score from the scores on the COVID-19 surveys. We calculated the change in anxiety by subtracting the T score on the pre–COVID-19 survey from the scores on the COVID-19 surveys. This was repeated at each survey wave for respondents who completed more than 1 wave. If participants had more than 1 WISDOM survey before COVID-19, the last survey before COVID-19 was used.

Change in anxiety and perceived breast cancer risk was estimated using paired 2-tailed *t* tests that compared participants’ responses from before COVID-19 to responses during COVID-19. We evaluated the relationship between perceived COVID-19 risk and change in perceived breast cancer risk and general anxiety using all 4 survey waves. These relationships were explored without adjustment and after adjusting using ordinary least squares regression for age (40-64 years and 65 years or older), race (White, Black, Asian, multiracial, American Indian or Alaska Native, Native Hawaiian or other Pacific Islander, or other race), and education (high school or less, some college or technical school, or college graduate or more) with standard errors clustered by respondent. We predicted missed medical appointments and cancer screening due to COVID-19 and plans to cancel medical care and cancer screening, across the 4 survey waves and from perceived COVID-19 risk in the same survey wave. These models using ordinary least squares regression adjusted for age, education, race, COVID-19 status, number of medical conditions, and survey wave through the inclusion of these variables as predictors in the regression model with standard errors clustered by respondent.

In order to explore whether perceived COVID-19 risk influences patients’ perceived risk of breast cancer, as well as their anxiety levels, we conducted a longitudinal analysis using a regression framework including a series of separate regression models. The first 2 models include the 6981 participants who completed 2 COVID-19 survey waves in a row and provided baseline assessments of anxiety and breast cancer risk. The first model included perceived COVID-19 risk, age, race, education, and wave. The second model added change in general anxiety between the prior survey wave and baseline in order to explore the influence between antecedent change in anxiety and the relationship between perceived COVID-19 risk and change in perceived breast cancer risk. The third model (N=16,311 because all cases are included) included a change in anxiety between the current survey wave and baseline. In a fourth model, we added the report during the current wave of canceled cancer screening during the past 2 months.

We repeated the analyses of the relationship between perceived COVID-19 risk and perceived breast cancer risk on the 1524 women who completed all 4 surveys. Because the results are similar to the full sample, the results are not described in the text but are displayed in Multimedia Appendix 1.

All analyses were performed on unweighted data. Binary outcomes (eg, cancellation of medical appointments) were analyzed using logistic regression, and continuous outcomes (eg, PROMIS4 anxiety scale) were analyzed using ordinary least squares regression. Analyses used R (version 4.1.2; R Foundation for Statistical Computing).

## Results

### Patient Sample and Characteristics

The 13,002 women who responded to at least 1 survey had a mean age of 58 years; 27% (n=3540) of women were aged 65 years or older, 84% (n=10,975) of women were White, and 76% (n=9898) of women graduated from college. A total of 64% (n=8298) of women reported no serious medical conditions and 47% (n=6120) of women reported no anxiety at baseline. There was little difference in demographic and clinical characteristics among respondents across waves (Table S2 in Multimedia Appendix 1). In pre–COVID-19 surveys, 9282 women provided general anxiety responses and 8839 provided perceived breast cancer risk responses.

### Perceived COVID-19 Risk, Breast Cancer Risk, and Anxiety

In their first survey response, 29.4% (n=3827) of respondents felt their COVID-19 risk over the next month was very low, 37.4% (n=4867) of respondents felt their risk was moderately low, 21.1% (n=2742) of respondents felt their risk was neither high nor low, 5.1% (n=659) of respondents felt their risk was moderately high, 0.5% (n=63) of respondents felt their risk was very high, 5.4% (n=704) of respondents did not provide a response, and 1.1% (n=140) of respondents were not asked the question because they had tested positive for COVID-19 (Table S2 in Multimedia Appendix 1).

Compared to before COVID-19, participants’ general anxiety was higher during the first COVID-19 survey completed (mean T score 53.5 vs 49.7 before COVID-19; mean difference 3.8, 95% CI 3.6-4.0; *P*<.001), but there was no overall difference in perceived breast cancer risk (mean 32.5% first COVID-19 survey vs 32.5% before COVID-19; mean difference 0.24, 95% CI –0.47 to 0.52; *P*=.93). Mean T scores by survey wave for general anxiety and perceived breast cancer risk were stable across waves (Table 1).

"During survey wave 1 (May-June 2020), 31% (2204/7186) of women reported that they had no general medical care scheduled. Of those with scheduled care over the prior 2 months, 83% (4112/4982) of women missed medical care. During that same period, 35% (1209/3426) of respondents with scheduled cancer screening missed the appointment. Projecting over the next 2 months, 17% (679/3987) of women planned to cancel general medical care, and 20% (547/2685) of women planned to cancel cancer screening.

**Table 1:** General anxiety and perceived breast cancer risk before and during COVID-19^a^.

Period	PROMIS4^b^ anxiety (range 40.3-81.6), mean T score (95% CI)	Perceived breast cancer risk (range 0-100), mean probability (%; 95% CI)
Before COVID-19	49.8 (49.7-50.0)	32.9 (32.5-33.4)
COVID-19 wave 1	53.7 (53.5-53.8)	31.9 (31.4-32.4)
COVID-19 wave 2	54.0 (53.8-54.2)	31.6 (31.1-32.2)
COVID-19 wave 3	53.3 (53.1-53.6)	31.3 (30.7-31.9)
COVID-19 wave 4	53.1 (52.9-53.3)	31.6 (31.0-32.2)

^a^Cell entries present unweighted averages with 95% CI in parentheses. The range of the PROMIS4 anxiety mean T-score is from 40.3 to 81.6 and the range of the perceived breast cancer risk is from 0% to 100%. Cell counts may vary by outcome measure as not all respondents answered each question. Data in the table differ slightly from the data presented in the text, which is a paired comparison. See also longitudinal cohort comparison in Table S5 in Multimedia Appendix 1. Baseline data are from the most recent response before COVID-19.

^b^PROMIS4: Patient-Reported Outcomes Measurement Information System 4-item.

### Relationship of Perceived COVID-19 Risk With Change in Anxiety, Change in Perceived Breast Cancer Risk, and Missed Medical Care

**Figure 1:**
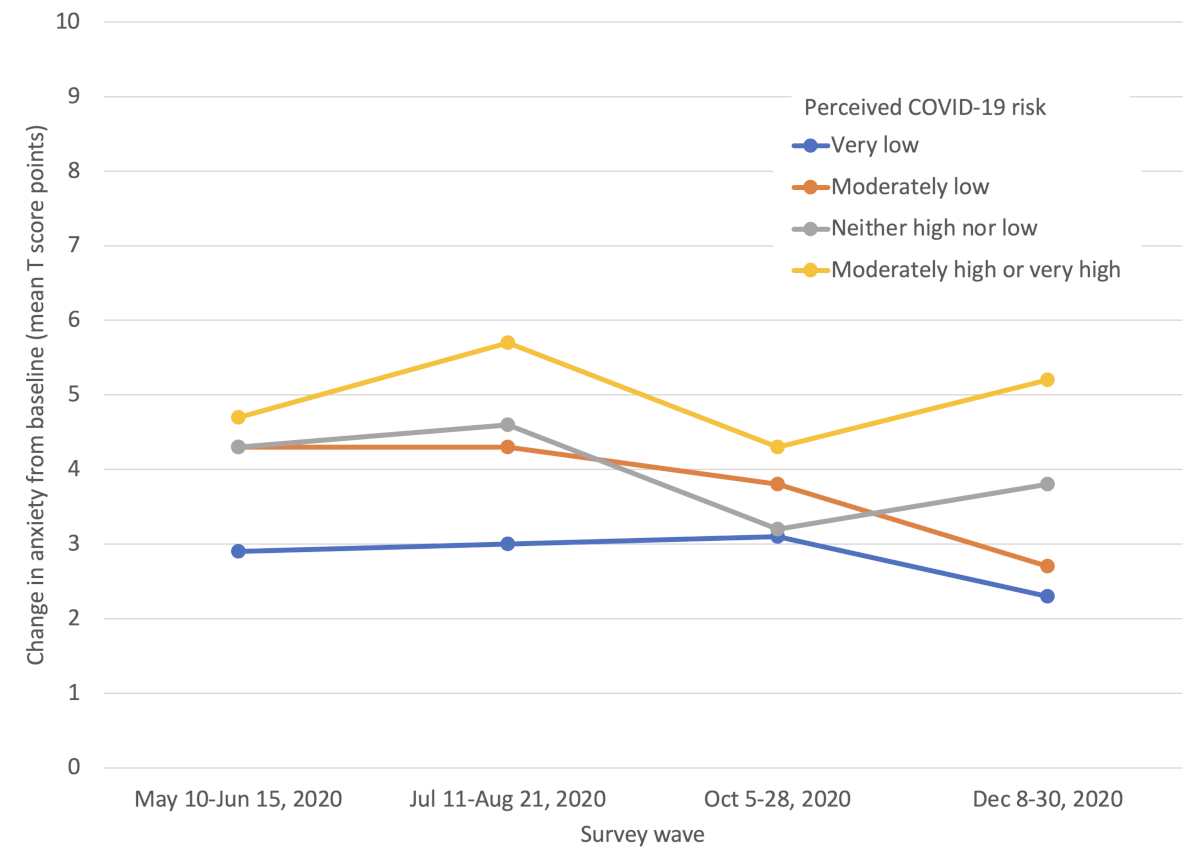
Change in general anxiety by perceived COVID-19 risk.

Higher perceived COVID-19 risk was associated with increased general anxiety across all survey waves. For example, in survey wave 4, a mean increase of 5.2, 3.8, 2.7, and 2.3 in PROMIS4 anxiety T score from baseline was noted for respondents with moderately or very high, neither high nor low, moderately low, and very low perceived COVID-19 risk, respectively (Figure 1 and Table 2).

**Figure 2:**
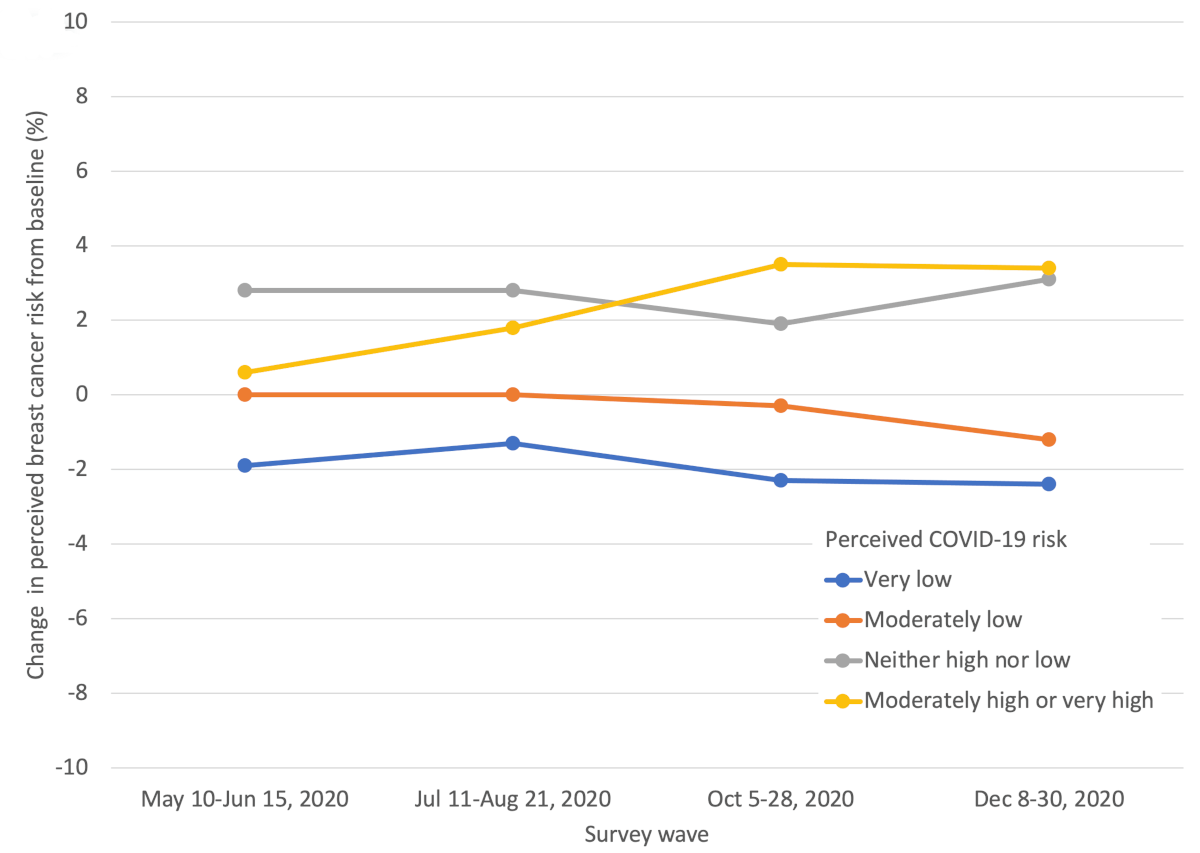
Change in perceived breast cancer risk by perceived COVID-19 risk.

Change in breast cancer risk perception had a more complex relationship with perceived COVID-19 risk. Perceived breast cancer risk decreased from pre–COVID-19 levels for people with very low perceived COVID-19 risk. For women with moderately low perceived COVID-19 risk, the pre- or postpandemic difference is not statistically distinguishable from zero. However, for women for whom the perceived COVID-19 risk was “neither high nor low” or perceived risk was moderately high or very high, perceived breast cancer risk increased during most survey waves. Survey wave 4 demonstrates this trend best: change in perceived breast cancer risk increased from –2.4 to –1.2 to +3.1 to +3.4 across the 4 levels of perceived COVID-19 risk from very low to moderately or very high, respectively (Figure 2 and Table 2).

After adjusting for age, race, education, and survey wave, both changes in general anxiety and change in perceived breast cancer risk remain statistically significantly, directly related to perceived COVID-19 risk. Compared to women with very low perceived COVID-19 risk, those with moderately low perceived COVID-19 risk increased 1.07 (95% CI 0.75-1.39) anxiety T score points from before COVID-19. This change was 1.26 (95% CI 0.86-1.66) for neither high nor low perceived COVID-19 risk and 2.38 (95% CI 1.67-3.09) for moderately or very high perceived COVID-19 risk. Concerning change in perceived breast cancer risk, after adjustment, compared to women with very low perceived COVID-19 risk, those with moderately low perceived COVID-19 risk increased 1.54% (95% CI 0.75%-2.33%) in perceived breast cancer risk; this increase was 4.28% (95% CI 3.30%-5.25%) for women with neither high nor low perceived COVID-19 risk and 3.67% (95% CI 1.94%-5.40%) for women with moderately or very high perceived COVID-19 risk (Table 3).

Perceived COVID-19 risk was not consistently associated with whether women missed medical care or cancer screening during the prior 2 months or planned to cancel medical care or cancer screening in the next 2 months, without (Table S3 in Multimedia Appendix 1) or with adjustment for age, education, race, COVID-19 infection status, number of medical diagnoses, and survey wave (Table S4 in Multimedia Appendix 1).

**Table 2:** Changes in general anxiety and perceived breast cancer risk by perceived COVID-19 risk^a^.

Change and wave			Perceived COVID-19 risk (next 30 days), mean T score (95% CI)									Total, n
			Very low		Moderately low		Neither high nor low		Moderately high or very high			
**Change in general anxiety (range 40.3-81.6)**		**n=5361**		**n=7368**		**n=4092**		**n=999**				
	Wave 1	2.9 (2.5 to 3.2)		4.3 (4.0 to 4.6)		4.3 (3.9 to 4.8)		4.7 (3.6 to 5.8)		6128		
	Wave 2	3.0 (2.6 to 3.5)		4.3 (4.0 to 4.7)		4.6 (4.1 to 5.0)		5.7 (4.7 to 6.7)		4772		
	Wave 3	3.1 (2.7 to 3.6)		3.8 (3.4 to 4.2)		3.2 (2.7 to 3.8)		4.3 (3.1 to 5.4)		3509		
	Wave 4	2.3 (1.8 to 2.8)		2.7 (2.3 to 3.1)		3.8 (3.2 to 4.3)		5.2 (4.1 to 6.2)		3411		
**Change in breast cancer risk (range 0-100)**		**n=4871**		**n=7121**		**n=3902**		**n=965**				
	Wave 1	–1.9 (–2.8 to –1.1)		0.0 (–0.7 to 0.8)		2.8 (1.7 to 3.9)		0.6 (–1.8 to 3.1)		5829		
	Wave 2	–1.3 (–2.3 to –0.2)		0.0 (–0.8 to 0.9)		2.8 (1.6 to 3.9)		1.8 (–0.8 to 4.3)		4505		
	Wave 3	–2.3 (–3.5 to –1.2)		–0.3 (–1.3 to 0.8)		1.9 (0.5 to 3.4)		3.5 (0.4 to 6.6)		3314		
	Wave 4	–2.4 (–3.7 to –1.0)		–1.2 (–2.1 to –0.2)		3.1 (1.6 to 4.5)		3.4 (1.0 to 5.9)		3211		

^a^Cell entries present the mean with 95% CI in parentheses for change in PROMIS4 (Patient-Reported Outcomes Measurement Information System 4) anxiety and perceived breast cancer risk from baseline to the survey wave for individuals who perceived a certain level of COVID-19 risk over the next 30 days in that wave. The survey was conducted in the following time periods: wave 1: from May 10 to June 15, 2020; wave 2: from July 11 to August 21, 2020; wave 3: from October 5 to 28, 2020; and wave 4: from December 8 to 30, 2020.

**Table 3:** Change in anxiety and change in perceived breast cancer risk with perceived COVID-19 risk after adjustment for patient characteristics and time^a^.

		Change in PROMIS4^b^ anxiety (range 40.3-81.6)	Change in perceived breast cancer risk (range 0-100)
**Perceived COVID-19 risk (30 days), OLS^c^ estimate (95% CI)**			
	Moderately low	1.07 (0.75 to 1.39)***	1.54 (0.75 to 2.33)***
	Neither high nor low	1.26 (0.86 to 1.66)***	4.28 (3.30 to 5.25)***
	Moderately or very high	2.38 (1.67 to 3.09)***	3.67 (1.94 to 5.40)***
**Age (years), OLS estimate (95% CI)**			
	≥65	0.39 (0.028 to 0.76)*	–1.26 (–2.16 to –0.37)**
**Race or ethnicity, OLS estimate (95% CI)**			
	Asian, Native Hawaiian, or other Pacific Islander	–0.025 (–1.01 to 0.96)	0.36 (–1.82 to 2.54)
	Black	0.40 (–0.92 to 1.71)	1.15 (–3.62 to 5.92)
	Multiracial	0.64 (–0.28 to 1.56)	0.64 (–1.61 to 2.89)
	Other, unknown, or American Indian or Alaskan Native	0.32 (–0.81 to 1.45)	–1.24 (–4.46 to 1.97)
**Education, OLS estimate (95% CI)**			
	College graduate or more	1.55 (1.11 to 1.99)***	0.65 (–0.43 to 1.73)
**Time fixed effects, OLS estimate (95% CI)**			
	Wave 2	0.14 (–0.098 to 0.37)	0.29 (–0.31 to 0.89)
	Wave 3	–0.47 (–0.74 to –0.21)**	–0.26 (–0.96 to 0.44)
	Wave 4	–1.053 (–1.33 to –0.77)**	–0.32 (–1.04 to 0.41)
**Intercept, OLS estimate (95% CI)**		1.70 (1.22 to 2.19)***	–1.81 (–3.00 to –0.63)**
N		17,466	16,524
*R^2^*		0.015	0.009
Adjusted* R^2^*		0.014	0.008
Akaike information criterion		121,852.0	144,696.7
Bayesian information criterion		121,960.8	144,804.7
Root mean square error		7.91	19.27
*F* test (*df*)		15.97 (12, 8493)	8.295 (12, 8055)

^a^*P* value thresholds are *P<.05, **P<.01, and ***P<.001. Ordinary least squares regression model predicting change in general anxiety T score and change in perceived breast cancer risk from perceived COVID-19 risk. SEs are clustered at the participant level. Reference categories: age 40-64 years; education: some college or technical school or less; race: White; perceived COVID-19 risk: very low; time: wave 1. Cell entries indicate the ordinary least squares estimate followed by the approximate 95% CI, in brackets, and the *P* value threshold, described above.

^b^PROMIS4: Patient-Reported Outcomes Measurement Information System 4-item.

^c^OLS: ordinary least squares.

### Exploring the Relationship Between Perceived COVID-19 Risk and Change in Perceived Breast Cancer Risk

We explored our hypotheses that higher perceived COVID-19 risk was associated with increased anxiety and canceled cancer screening that, in turn, led to increased perceived breast cancer risk. Table 4 shows the series of regression models predicting change in perceived breast cancer risk. Column 1, which includes a restricted cohort of 6981 women who completed surveys in 2 adjacent waves, shows that after accounting for age, race, education, and survey wave, compared to women with very low perceived COVID-19 risk, women with neither high nor low and moderately or very high perceived COVID risk had increases in perceived breast cancer risk of 3.47% (95% CI 2.08%-4.87%) and 5.40% (95% CI 2.87%-7.93%), respectively. To investigate whether these results are driven by increased anxiety during the COVID-19 pandemic, column 2 adds the change in general anxiety from before COVID-19 to the prior survey wave. This regression model, which avoids some issues of simultaneous measurement, demonstrates that prior wave change in general anxiety from baseline has virtually no impact on change in perceived breast cancer risk. The next model includes changes in anxiety from before the pandemic to the current survey wave (analogous to the change in breast cancer risk) and finds a weak statistically significant relationship with change in perceived breast cancer risk. However, the relationship between perceived breast cancer risk and perceived COVID-19 risk is only slightly altered (column 3). Finally, in column 4, patient cancellation of cancer screening in the prior 2 months was unrelated to the change in perceived breast cancer risk and does not appear to drive the relationship with perceived COVID-19 risk. Taken together, these models suggest that higher perceived COVID-19 risk is related to an increase in perceived breast cancer risk that is not mediated by a change in anxiety or missed cancer screening. When perceived COVID-19 risk was removed from the model in column 2, the effect on the relationship between anxiety and change in perceived breast cancer risk was minimal (data not shown).

**Table 4:** Relationship of change in perceived breast cancer risk to perceived COVID-19 risk while controlling for change in anxiety and missed cancer screening^a^.

			Change in perceived breast cancer risk						
			Restricted sample		Restricted sample (model includes change in general anxiety from prior survey wave)		Full sample (model includes change in general anxiety from current survey wave)		Full sample (model includes self-reported missed cancer screening)
**Perceived COVID-19 infection risk (30 days), OLS^b^ estimate (95% CI)**									
	Moderately low	0.513 (–0.649 to 1.676)		0.503 (–0.660 to 1.666)		1.482 (0.685 to 2.279)***		1.522 (0.729 to 2.314)***	
	Neither high nor low	3.473 (2.077 to 4.869)***		3.463 (2.065, 4.861) ***		4.170 (3.190 to 5.151)***		4.252 (3.277 to 5.228)***	
	Moderately or very high	5.399 (2.872 to 7.926)***		5.378 (2.851 to 7.905)***		3.510 (1.761 to 5.260)***		3.676 (1.945 to 5.407)***	
**Change in PROMIS4^c^anxiety scale T score, OLS estimate (95% CI)**									
	Previous wave	Not included		0.019 (–0.051 to 0.090)		—^d^		—	
	Current wave	Not included		—		0.060 (0.011 to 0.110)*		—	
**Cancer screening cancellations, OLS estimate (95% CI)**									
	Yes	Not in model		—		—		0.580 (–0.496 to 1.656)	
	Nothing scheduled or not sure	—		—		—		–0.015 (–0.709 to 0.679)	
**Age (years), OLS estimate (95% CI)**									
	≥65	–1.301 (–2.525 to –0.076)*		–1.307 (–2.531 to –0.084)*		–1.366 (–2.267 to –0.465)**		–1.310 (–2.209 to –0.411)**	
**Race and ethnicity, OLS estimate (95% CI)**									
	Asian, Native Hawaiian, or other Pacific Islander	–0.418 (–3.647 to 2.810)		–0.399 (–3.625 to –2.827)		0.396 (–1.802 to 2.594)		0.340 (–1.832 to 2.512)	
	Black	–2.076 (–11.049 to 6.898)		–2.085 (–11.066 to 6.896)		0.893 (–4.055 to 5.842)		0.943 (–3.849 to 5.736)	
	Multiracial	1.601 (–1.744 to 4.946)		1.582 (–1.761 to 4.925)		0.579 (–1.679 to 2.837)		0.613 (–1.639 to 2.865)	
	Other, unknown, or American Indian or Alaskan Native	–0.972 (–6.367 to 4.423)		–0.979 (–6.373 to 4.415)		–1.228 (–4.541 to 2.086)		–1.302 (–4.535 to 1.931)	
**Education, OLS estimate (95% CI)**									
	College graduate or more	0.020 (–1.501 to 1.542)		–0.007 (–1.530 to 1.515)		0.553 (–0.526 to 1.633)		0.623 (–0.457 to 1.702)	
**Time fixed effect**									
	Wave 2	—		—		0.272 (–0.326 to 0.869)		0.327 (–0.271 to 0.924)	
	Wave 3	–0.362 (–1.137 to 0.412)		–0.356 (–1.131 to 0.418)		–0.212 (–0.915 to 0.490)		–0.229 (–0.933 to 0.474)	
	Wave 4	–0.701 (–1.624 to 0.223)		–0.688 (–1.611 to 0.236)		–0.283 (–1.011 to 0.446)		–0.266 (–0.993 to 0.462)	
**Intercept, OLS estimate (95% CI)**			–0.341 (–2.051 to 1.369)		–0.389 (–2.113 to 1.334)		–1.881 (–3.074 to –0.688)**		–1.858 (–3.125 to –0.592)**
N			6,981		6,981		16,311		16,476
*R^2^*			0.01		0.011		0.01		0.009
Adjusted *R^2^*			0.009		0.009		0.009		0.008
Akaike information criterion			60717.2		60718.8		142749.4		144249.9
**Bayesian Information Criterion**			60806.3		60814.7		142864.9		144373.3
Root mean square error			18.68		18.68		19.22		19.26
*F* test (*df*)			4.427 (11, 3804)		4.101 (12, 3804)		8.095 (13, 7959)		7.249 (14, 8041)

^a^Ordinary least squares regression model predicting change in perceived breast cancer risk from perceived COVID-19 risk. Columns 1 and 2 include the restricted sample of women (n=6981) who completed 2 adjacent survey waves. Column 1 includes age, race, education, wave as a fixed effect, and perceived COVID-19 risk from the current wave. Column 2 adds the change in general anxiety between the prior wave and baseline. Column 3 adds to the column 1 model the change in general anxiety between the current wave and baseline. Column 4 adds to the column 1 model missed cancer screening in the prior 2 months. Reference categories: age 40-64 years; education: some college or technical school or less; race: White; perceived COVID-19 risk: very low; time: wave 1. Cell entries indicate the ordinary least squares estimate followed by the approximate 95% CI, in brackets, and the *P* value threshold as follows: *P<.05, **P<.01, and ***P<.001.

^b^OLS: ordinary least squares.

^c^PROMIS4: Patient-Reported Outcomes Measurement Information System 4-item.

^d^Not applicable.

## Discussion

### Principal Findings and Implications

Prospectively collected information compared with data collected during the COVID-19 pandemic provides insight into the magnitude and direction of the effect of a new risk on the ongoing perception of risk of another clinical condition and general anxiety. This is an unstudied issue that could have broad implications for at-risk individuals who have reason to engage in preventive behavior and are newly confronted by an unrelated threat. This study shows that the perceived risk of COVID-19 is associated with changes in the perceived risk of breast cancer. While it appeared overall that perceived breast cancer risk was unchanged before or after COVID-19, these data hid a clear dose-response relationship with perceived COVID-19 risk ranging from a 2% decrease among those with very low perceived COVID-19 risk to a 3% increase among those with highest perceived COVID-19 risk. This magnitude of change is about half that seen with a breast cancer risk feedback intervention [16]. While the implications of this change in breast cancer risk perception remain to be elucidated, perceived breast cancer risk is linked reliably, albeit not strongly, with early detection and preventive behavior [17,18]. Perceived risk of a potentially threatening condition is a key determinant of health behavior [10,19]. Demonstration that a new threat (eg, COVID-19) alters other health risk perceptions could have implications for disease prevention.

The mechanism by which COVID-19 perceived risk affects breast cancer perceived risk is unclear. Neither increase in anxiety nor missed cancer screening—both hypothesized to relate to the change in breast cancer risk perception—appear to play a role. Risk perception is complex with cognitive and emotional underpinnings [20,21]. Perceived risk of a clinical condition relates to family or genetic factors, salience [22], and anxiety, among others. Factors underlying perceived COVID-19 risk also include availability [23], gender [24], age [25], anxiety [26], and attention to and trust in the media [27]. While information about breast cancer risk and salience should not have been affected by COVID-19, risk perception is also influenced by contextual factors, such as the immediacy of a threat [28], perceived level of control, and fear [29]. These constructs may have affected both perceived COVID-19 risk and breast cancer risk. More exploration is needed into the linkage between change in breast cancer risk perception and COVID-19 risk perception.

This study confirms that COVID-19 was associated with an increase in general anxiety among a large cohort of middle-aged women, the majority of who did not have anxiety at baseline. General anxiety increased by one to two times the minimally important difference in the PROMIS4 measure, depending on perceived COVID-19 risk, and did not change appreciably over the first year of COVID-19. This confirms what a small number of pre– or post–COVID-19 longitudinal studies have found [30-32] and provides insight into the degree of anxiety and relationship to perceived COVID-19 risk.

### Limitations

Several factors limit the generalizability of these findings. Data are derived from women in a select age range who enrolled in a trial of breast cancer prevention. There was limited variation in race and education. Younger participants, who sustained greater emotional effects of COVID-19 [33], are not included. Moreover, the levels of change in anxiety and perceived breast cancer risk were modest and we have no preventive behavior data to link with changes in perceived breast cancer risk. Finally, while perceptions of COVID-19 risk are related to the perceived risk of breast cancer in substantively and statistically significant ways, we note that COVID-19 risk perceptions do not explain much of the variation in perceptions of breast cancer risk. This is likely because many things affect a person’s beliefs about their likelihood of being diagnosed with breast cancer and we do not measure all of those things here. While perceptions of COVID-19 risk are but one of many things associated with breast cancer risk perceptions—and do not drive these perceptions as much as other factors not measured here—the link between these 2 outcomes, one longstanding and the other novel, provides some insight into how patients connect disparate health risks.

### Conclusions

COVID-19 affected the perceived risk of developing breast cancer. This natural experiment merits experimental replication because the implications for disease prevention are formidable. The effect of a global threat, such as COVID-19, may have broad implications for health beyond the pandemic. These findings suggest that attention to preventive health behaviors will be needed as COVID-19 becomes endemic.

## Appendix

Multimedia Appendix 1 Supplementary tables.

## Abbreviations

PROMIS: Patient-Reported Outcome Measurement Information System
WISDOM: Women Informed to Screen Depending On Measures of Risk

## Data Availability

The data analyzed during this study are not publicly available because the study is ongoing but are available from the corresponding author upon reasonable request.
