# Supplementary material for: Relationship Between Perceived COVID-19 Risk and Change in Perceived Breast Cancer Risk: Prospective Observational Study
Source: JMIR Cancer. 2024 Dec 2;10:e47856. doi: 10.2196/47856 (PMC11650083; doi:10.2196/47856)
Supplement: Multimedia Appendix 1 [file cancer_v10i1e47856_app1.docx]

Table S1. Survey Sample and Completion Rate

| **Wave** | **Dates** | **Invited** | **Completed** |
| --- | --- | --- | --- |
| 1 | May 10 – June 15, 2020 | 25,588 | 7523 (29%) |
| 2 | July 11 – August 21, 2020 | 25,162 | 5849 (23%) |
| 3 | October 5 – October 28, 2020 | 27,576 | 5275 (19%) |
| 4 | December 8 – December 30, 2020 | 28,758 | 5774 (20%) |

Table S2. Socio-demographics of WISDOM subjects who responded to COVID-19 surveys

|  |  | All Subjects | Subject Cohorts | | Entire Sample by Wave | | | |
| --- | --- | --- | --- | --- | --- | --- | --- | --- |
| Variable | Level | - | Longitudinal | Cross-Sectional | Wave 1 | Wave 2 | Wave 3 | Wave 4 |
|  |  | N=13,002 | N=1,524 | N=11,478 | N=7,523 | N=5,849 | N=5,275 | N=5,774 |
| Age | 40-64 | 9,460 (72.8) | 899 (59.0) | 8,561 (74.6) | 5,266 (70.0) | 3,948 (67.6) | 3,520 (66.7) | 3,928 (68.0) |
|  | 65+ | 3,540 (27.2) | 625 (41.0) | 2,915 (25.4) | 2,257 (30.0) | 1,891 (32.4) | 1,754 (33.3) | 1,846 (32.0) |
| Race/Ethnicity | White | 10,975 (84.4) | 1,394 (91.5) | 9,581 (83.5) | 6,410 (85.2) | 4,974 (85.2) | 4,636 (87.9) | 5,073 (87.8) |
|  | Black or African American | 205 (1.6) | 20 (1.3) | 185 (1.6) | 90 (1.2) | 70 (1.2) | 80 (1.5) | 101 (1.7) |
|  | Asian | 510 (3.9) | 45 (3.0) | 465 (4.1) | 278 (3.7) | 223 (3.8) | 207 (3.9) | 199 (3.4) |
|  | American Indian or Alaska Native | 25 (0.2) | 1 (0.1) | 24 (0.2) | 8 (0.1) | 11 (0.2) | 9 (0.2) | 13 (0.2) |
|  | Native Hawaiian or Other Pacific Islander | 18 (0.1) | - | 18 (0.2) | 11 (0.1) | 4 (0.1) | 6 (0.1) | 7 (0.1) |
|  | Multi-racial | 486 (3.7) | 41 (2.7) | 445 (3.9) | 245 (3.3) | 188 (3.2) | 195 (3.7) | 225 (3.9) |
|  | Other race | 206 (1.6) | 9 (0.6) | 197 (1.7) | 103 (1.4) | 93 (1.6) | 71 (1.3) | 58 (1.0) |
|  | No Data | 577 (4.4) | 14 (0.9) | 563 (4.9) | 378 (5.0) | 276 (4.7) | 71 (1.3) | 99 (1.7) |
| Education | High school or less | 340 (2.6) | 39 (2.6) | 301 (2.6) | 187 (2.5) | 148 (2.5) | 144 (2.7) | 157 (2.7) |
|  | Some college or technical school | 2,234 (17.2) | 241 (15.8) | 1,993 (17.4) | 1,256 (16.7) | 984 (16.9) | 918 (17.4) | 971 (16.8) |
|  | College graduate or more | 9,898 (76.1) | 1,235 (81.0) | 8,663 (75.5) | 5,728 (76.1) | 4,451 (76.2) | 4,164 (78.9) | 4,573 (79.2) |
|  | No Data | 530 (4.1) | 9 (0.6) | 521 (4.5) | 352 (4.7) | 256 (4.4) | 49 (0.9) | 74 (1.3) |
| # Serious Diagnoses | 0 | 8,298 (63.8) | 905 (59.4) | 7,393 (64.4) | 4,745 (63.1) | 3,666 (62.8) | 3,260 (61.8) | 3,659 (63.4) |
|  | 1 | 3,286 (25.3) | 430 (28.2) | 2,856 (24.9) | 1,951 (25.9) | 1,469 (25.2) | 1,395 (26.4) | 1,535 (26.6) |
|  | 2 or more | 1,418 (10.9) | 189 (12.4) | 1,229 (10.7) | 827 (11.0) | 704 (12.1) | 620 (11.8) | 581 (10.1) |
| COVID Risk (Month) | Very Low | 3,827 (29.4) | 511 (33.5) | 3,316 (28.9) | 2,407 (32.0) | 1,527 (26.2) | 1,537 (29.1) | 1,398 (24.2) |
|  | Moderately Low | 4,867 (37.4) | 635 (41.7) | 4,232 (36.9) | 2,867 (38.1) | 2,357 (40.4) | 2,111 (40.0) | 2,202 (38.1) |
|  | Neither High or Low | 2,742 (21.1) | 300 (19.7) | 2,442 (21.3) | 1,563 (20.8) | 1,362 (23.3) | 1,126 (21.3) | 1,340 (23.2) |
|  | Moderately High | 659 (5.1) | 60 (3.9) | 599 (5.2) | 322 (4.3) | 299 (5.1) | 259 (4.9) | 377 (6.5) |
|  | Very High | 63 (0.5) | 7 (0.5) | 56 (0.5) | 27 (0.4) | 20 (0.3) | 22 (0.4) | 45 (0.8) |
|  | Not Asked* | 140 (1.1) | 6 (0.4) | 134 (1.2) | 27 (0.4) | 33 (0.6) | 66 (1.3) | 191 (3.3) |
|  | No Data | 704 (5.4) | 5 (0.3) | 699 (6.1) | 310 (4.1) | 241 (4.1) | 154 (2.9) | 222 (3.8) |
| General Anxiety (PROMIS4) | Normal Anxiety | 6,120 (47.1) | 831 (54.5) | 5,289 (46.1) | 3,690 (49.0) | 2,771 (47.5) | 2,684 (50.9) | 3,020 (52.3) |
|  | Mild Anxiety | 3,619 (27.8) | 416 (27.3) | 3,203 (27.9) | 2,065 (27.4) | 1,692 (29.0) | 1,477 (28.0) | 1,583 (27.4) |
|  | Moderate Anxiety | 2,294 (17.6) | 257 (16.9) | 2,037 (17.7) | 1,329 (17.7) | 1,019 (17.5) | 856 (16.2) | 846 (14.6) |
|  | Severe Anxiety | 223 (1.7) | 16 (1.0) | 207 (1.8) | 101 (1.3) | 97 (1.7) | 84 (1.6) | 97 (1.7) |
|  | No data | 746 (5.7) | 4 (0.3) | 742 (6.5) | 338 (4.5) | 260 (4.5) | 174 (3.3) | 229 (4.0) |

Longitudinal cohort includes 1524 women who completed all 4 surveys. Cross-sectional cohort includes 11,478 women who completed 1 to 3 surveys.

* 1.1% not asked the question because they had tested positive for COVID.

Table S3. Cancellation of Medical Appointments by Level of Perceived COVID-19 Risk

(May - June 2020)

|  | **Percent of Medical Appointments Not Scheduled or Missed or Cancelled (% and Confidence Interval)** | | | | |  |
| --- | --- | --- | --- | --- | --- | --- |
|  | **Perceived COVID-19 Risk (Next 30 Days)** | | | | |  |
| **Cancelled Medical Appointments** | **Very Low**  **(N=2407)** | **Moderately Low (N=2867)** | **Neither High or Low (N=1563)** | **Moderately or Very High (N=349)** |  |  |
| **Past two months** |  |  |  |  |  |  |
| General appointment not scheduled | 31.2 (29.3,33.0) | 29.9 (28.3,31.6) | 31.4 (29.1,33.7) | 30.1 (25.3,34.9) |  |  |
| General appointment missed or cancelled * | 81.8 (80.0,83.7) | 83.4 (81.8,85.1) | 82.9 (80.7,85.2) | 78.3 (73.1,83.5) |  |  |
| Cancer screenings missed or cancelled* | 34.7 (31.9,37.5) | 37.2 (34.6,39.8) | 33.9 (30.5,37.3) | 30.3 (23.6,36.9) |  |  |
| **Future two months** |  |  |  |  |  |  |
| General appointment missed or cancelled | 18.9 (16.7,21.0) | 17.8 (15.9,19.7) | 13.9 (11.6,16.2) | 13.2 (8.6,17.8) |  |  |
| Cancer screenings missed or cancelled | 20.6 (18.0,23.3) | 22.5 (20.0,25.0) | 16.7 (13.6,19.8) | 17.5 (11.3,23.7) |  |  |
| **Note:** Cells present percentages with 95% confidence intervals in parentheses.  * Excludes patients with no appointment scheduled | | | | | | |

Table S4. Relationship of Missed Medical Appointments to Perceived COVID-19 Infection Risk after Adjustment for Patient Characteristics, COVID-19 status, Number of Diagnoses and Time

|  | **Past Two Months** | | | **Next Two Months** | |
| --- | --- | --- | --- | --- | --- |
|  | Nothing Scheduled | General Medical Care Appointment Missed or Cancelled | Cancer Screening Missed or Cancelled | Plan to Cancel  General Medical Care Appointment | Plan to Cancel Cancer Screening |
| **Age** |  |  |  |  |  |
| 65+ years old | 0.99 [0.99, 0.99] *** | 1.00 [1.00, 1.01] | 1.00 [0.99, 1.00] | 1.00 [1.00, 1.01] | 1.01 [1.00, 1.02] * |
| **Education** |  |  |  |  |  |
| Some college or technical school | 0.87 [0.69, 1.09] | 1.11 [0.83, 1.47] | 1.12 [0.79, 1.59] | 1.35 [0.79, 2.29] | 1.91 [1.03, 3.54] * |
| College graduate or more | 0.64 [0.51, 0.80] *** | 1.26 [0.96, 1.66] | 1.39 [0.99, 1.93] | 2.18 [1.32, 3.61] ** | 2.63 [1.45, 4.77] ** |
| **Race** |  |  |  |  |  |
| Black | 1.15 [0.87, 1.52] | 0.96 [0.68, 1.34] | 0.87 [0.56, 1.36] | 0.93 [0.55, 1.59] | 1.06 [0.63, 1.80] |
| AAPI | 1.05 [0.88, 1.25] | 1.23 [1.01, 1.50] * | 1.23 [0.94, 1.61] | 1.34 [0.98, 1.83] | 1.79 [1.25, 2.56] ** |
| Multi-racial | 0.83 [0.69, 1.00] | 1.17 [0.95, 1.45] | 1.19 [0.92, 1.54] | 1.26 [0.92, 1.71] | 1.28 [0.91, 1.81] |
| Other/Unknown/AIAN | 1.18 [0.95, 1.47] | 1.16 [0.86, 1.58] | 1.44 [1.05, 1.99] * | 1.03 [0.65, 1.64] | 1.13 [0.70, 1.82] |
| **COVID-19 Status** |  |  |  |  |  |
| Believes Had COVID | 0.86 [0.71, 1.05] | 1.38 [1.07, 1.77] * | 1.28 [1.00, 1.65] | 0.89 [0.63, 1.26] | 0.98 [0.69, 1.40] |
| Believes Household (Not  Self) Had COVID | 0.82 [0.61, 1.11] | 1.33 [0.91, 1.93] | 0.96 [0.61, 1.49] | 1.00 [0.56, 1.75] | 0.75 [0.39, 1.46] |
| Tested Positive for COVID | 1.13 [0.86, 1.47] | 2.69 [1.97, 3.68] *** | 1.53 [1.02, 2.28] * | 0.67 [0.36, 1.27] | 0.50 [0.23, 1.11] |
| **Count of Diagnoses (0 - 6)** | 0.65 [0.61, 0.68] *** | 1.07 [1.01, 1.12] * | 1.04 [0.98, 1.10] | 1.04 [0.96, 1.12] | 1.02 [0.93, 1.11] |
| **Perceived COVID Risk**  **(30 Days, Categorical)** |  |  |  |  |  |
| Moderately Low | 0.94 [0.87, 1.02] | 1.06 [0.97, 1.16] | 1.11 [0.99, 1.24] | 0.96 [0.84, 1.10] | 1.05 [0.90, 1.22] |
| Neither High or Low | 0.97 [0.89, 1.07] | 1.05 [0.95, 1.17] | 0.98 [0.86, 1.12] | 0.80 [0.67, 0.94] ** | 0.77 [0.63, 0.94] ** |
| Moderately/Very High | 0.99 [0.86, 1.15] | 1.22 [1.03, 1.46] * | 1.02 [0.83, 1.25] | 1.06 [0.82, 1.37] | 1.17 [0.88, 1.56] |
| **Time Fixed Effects** |  |  |  |  |  |
| Wave 2 | 0.91 [0.85, 0.98] * | 0.30 [0.28, 0.33] *** | 0.86 [0.78, 0.95] ** | 0.52 [0.45, 0.59] *** | 0.62 [0.54, 0.73] *** |
| Wave 3 | 0.82 [0.76, 0.89] *** | 0.08 [0.07, 0.09] *** | 0.44 [0.39, 0.49] *** | 0.22 [0.18, 0.26] *** | 0.34 [0.28, 0.40] *** |
| Wave 4 | 0.86 [0.79, 0.93] *** | 0.09 [0.08, 0.10] *** | 0.46 [0.41, 0.52] *** | 0.50 [0.43, 0.57] *** | 0.55 [0.47, 0.64] *** |
| (Intercept) | 1.41 [1.04, 1.92] * | 2.81 [1.93, 4.11] *** | 0.49 [0.31, 0.79] ** | 0.08 [0.04, 0.16] *** | 0.06 [0.03, 0.13] *** |
| Log-likelihood | -13018.3 | -9169.9 | -6337.6 | -4347.9 | -3368.7 |
| AIC | 26072.7 | 18375.9 | 12711.2 | 8731.7 | 6773.4 |
| BIC | 26216.9 | 18514.1 | 12843 | 8867.2 | 6900.5 |
| N | 22,311 | 15,966 | 11,211 | 13,707 | 8,607 |

**Note:** *P*-value thresholds are **P*<.05, ***P<*.01 and ****P<*.0.001. Cell entries present odds ratios calculated by exponentiating the logistic regression coefficients. Standard errors are HC2 standard errors clustered at the subject level

AIAN=American Indian or Alaskan Native. AAPI=Asian, Native Hawaiian or Other Pacific Islander.

Reference categories: Age 40-64, Education High school or less, Race White, COVID-19 status Never had COVID-19, Perceived COVID-19 risk Very low, Time Wave 1.

Table S5. Longitudinal cohort: Changes in General Anxiety and Perceived Breast Cancer Risk before and during COVID-19

|  | **PROMIS4 Anxiety** | **Perceived Breast Cancer Risk** |
| --- | --- | --- |
| *Period* | *Mean T-Score (40.3 - 81.6)* | *Mean Probability (0 - 100)* |
| Baseline | 49.2 (48.7, 49.6) | 30.2 (29.0, 31.4) |
| Wave 1 | 53.1 (52.7, 53.5) | 30.7 (29.5, 31.8) |
| Wave 2 | 52.8 (52.4, 53.3) | 30.3 (29.1, 31.5) |
| Wave 3 | 52.4 (52.0, 52.8) | 30.1 (28.9, 31.3) |
| Wave 4 | 51.7 (51.3, 52.2) | 30.5 (29.3, 31.6) |
| Count | 1,331 | 1,241 |

**Note:** Cell entries present unweighted averages with 95% confidence intervals in parentheses. Baseline data are from the most recent response before COVID-19. Only respondents who had baseline data from before COVID-19 and who completed the measure in every survey wave are included.

Table S6. Longitudinal cohort: Changes in General Anxiety and Perceived Breast Cancer Risk by Perceived COVID-19 Risk

|  | **Perceived COVID-19 Risk (Next 30 Days)** | | | | | N |  |
| --- | --- | --- | --- | --- | --- | --- | --- |
| **Change in General Anxiety**  (40.3 – 81.6) | Very Low | Moderately Low | Neither High  or Low | Moderately High/  Very High |  | |  |
| Wave 1 | 3.4 (2.7,4.1) | 4.1 (3.5,4.8) | 4.7 (3.7,5.8) | 2.9 (0.5,5.3) | 1350 | |  |
| Wave 2 | 3.0 (2.3,3.8) | 3.5 (2.9,4.1) | 4.4 (3.4,5.3) | 6.6 (4.5,8.6) | 1350 | |  |
| Wave 3 | 3.1 (2.3,3.9) | 3.4 (2.8,4.0) | 3.0 (2.1,3.9) | 4.1 (2.1,6.1) | 1343 | |  |
| Wave 4 | 1.8 (0.9,2.6) | 2.3 (1.7,2.9) | 3.6 (2.8,4.4) | 4.1 (2.3,5.9) | 1308 | |  |
| N | 1542 | 2311 | 1215 | 283 |  | |  |
| **Change in Breast Cancer Risk**  (0 – 100) |  |  |  |  |  | |  |
| Wave 1 | -1.5 (-3.2,0.2) | 1.1 (-0.4,2.6) | 1.0 (-1.4,3.4) | 0.5 (-5.5,6.5) | 1282 | |  |
| Wave 2 | -2.2 (-4.0,-0.5) | -0.4 (-1.9,1.1) | 2.9 (0.9,4.9) | 4.0 (-1.6,9.5) | 1276 | |  |
| Wave 3 | -1.7 (-3.5,0.1) | -0.1 (-1.7,1.5) | 0.3 (-2.0,2.6) | 3.8 (-1.2,8.7) | 1271 | |  |
| Wave 4 | -0.8 (-2.7,1.2) | -1.6 (-3.0,-0.1) | 2.4 (0.2,4.6) | 3.5 (-0.7,7.6) | 1233 | |  |
| N | 1416 | 2216 | 1159 | 271 |  | |  |
| **Note:** Cell entries present the change in PROMIS4 anxiety and perceived breast cancer risk from baseline to the survey wave for individuals who perceived a certain level of COVID-19 risk over the next 30 days in that wave. Confidence intervals are in parentheses. | | | | | | | |

Table S7. Longitudinal cohort: Relationship of Change in Anxiety and Change in Perceived Breast Cancer Risk to Perceived COVID-19 Risk

|  | **Change in PROMIS4 Anxiety** (40.3 - 81.6) | **Change in Perceived Breast Cancer Risk** (0 - 100) |
| --- | --- | --- |
| **Perceived COVID-19 Risk (30 Days)** |  |  |
| Moderately low | 0.436 [-0.180, 1.053] | 1.240 [-0.187, 2.667] |
| Neither high nor low | 1.083 [ 0.252, 1.913] * | 3.105 [ 1.257, 4.953] ** |
| Moderately/Very high | 1.567 [ 0.064, 3.069] * | 4.325 [ 0.455, 8.196] * |
| **Age** |  |  |
| 65 years old and older | 0.306 [-0.438, 1.050] | -1.168 [-2.917, 0.580] |
| **Race** |  |  |
| Black | 0.578 [-2.113, 3.270] | 1.155 [-12.810, 15.119] |
| Multi-racial | 0.776 [-1.519, 3.071] | -0.186 [-5.527, 5.156] |
| AAPI | -2.636 [-5.618, 0.347] | -1.635 [-6.871, 3.601] |
| Other/Unknown/AIAN | 3.755 [ 0.594, 6.916] * | 0.835 [-10.460, 12.130] |
| **Education** |  |  |
| College graduate or more | 2.088 [ 1.111, 3.066] *** | 0.360 [-1.934, 2.654] |
| **Time Fixed Effects** |  |  |
| Wave 2 | -0.296 [-0.631, 0.038] + | -0.203 [-1.055, 0.650] |
| Wave 3 | -0.754 [-1.106, -0.402] *** | -0.479 [-1.398, 0.440] |
| Wave 4 | -1.446 [-1.835, -1.058] *** | -0.437 [-1.392, 0.519] |
| (Intercept) | 1.672 [ 0.617, 2.726] ** | -0.962 [-3.431, 1.508] |
| N | 5,340 | 5,049 |
| R-Squared | 0.022 | 0.007 |
| Adj. R-Squared | 0.02 | 0.004 |
| AIC | 37,189.7 | 43,817.0 |
| BIC | 37,281.8 | 43,908.4 |
| RMSE | 7.86 | 18.48 |
| F-Test | F(12, 1357) = 7.434 | F(12, 1290) = 1.345 |

**Note:** *P*-value thresholds are **P*<.05, ***P<*.01 and ****P<*.0.001

Ordinary least squares regression model predicting change in change in general anxiety T score and change in perceived breast cancer risk from perceived COVID-19 risk. Standard errors are clustered at the subject level.

AIAN=American Indian or Alaskan Native. AAPI=Asian, Native Hawaiian or Other Pacific Islander.

Reference categories: Age 40-64, Education High school or less, Race White, Perceived COVID-19 risk Very low, Time Wave 1.

Table S8. Longitudinal Cohort: Relationship of Change in Perceived Breast Cancer Risk to Perceived COVID-19 Risk while Controlling for Change in Anxiety and Missed Cancer Screenings

|  | **Change in Perceived Breast Cancer Risk** | | | |
| --- | --- | --- | --- | --- |
|  | Restricted sample | Restricted sample. Model includes change in general anxiety from prior survey wave | Full sample. Model includes change in general anxiety from current survey wave | Full sample. Model includes self-reported missed cancer screening |
| **Perceived COVID-19**  **Infection Risk (30 Days)** |  |  |  |  |
| Moderately Low | 0.864 [-0.749, 2.478] | 0.860 [-0.752, 2.473] | 1.184 [-0.242, 2.610] | 1.286 [-0.146, 2.719] + |
| Neither High or Low | 3.354 [ 1.395, 5.312] *** | 3.348 [ 1.387, 5.309] *** | 2.983 [ 1.126, 4.840] ** | 3.129 [ 1.275, 4.984] *** |
| Moderately/Very High | 5.311 [ 1.457, 9.164] ** | 5.291 [ 1.441, 9.140] ** | 4.320 [ 0.417, 8.223] * | 4.340 [ 0.467, 8.213] * |
| **Change in PROMIS4**  **Anxiety Scale T-Score** |  |  |  |  |
| Previous Wave |  | 0.027 [-0.078, 0.132] |  |  |
| Current Wave |  |  | 0.037 [-0.066, 0.139] |  |
| **Cancer Screening**  **Cancellations** |  |  |  |  |
| Yes |  |  |  | 0.567 [-1.621, 2.754] |
| Nothing Scheduled  or Not Sure |  |  |  | -0.232 [-1.501, 1.038] |
| **Age** |  |  |  |  |
| 65 Years old and older | -1.040 [-2.840, 0.760] | -1.050 [-2.850, 0.750] | -1.312 [-3.063, 0.439] | -1.199 [-2.948, 0.551] |
| **Race** |  |  |  |  |
| Black | -1.744 [-17.696, 14.208] | -1.779 [-17.751, 14.194] | 0.110 [-15.043, 15.263] | 1.166 [-12.774, 15.105] |
| Multi-racial | -0.203 [-5.154, 4.748] | -0.236 [-5.177, 4.705] | -0.191 [-5.516, 5.134] | -0.271 [-5.643, 5.101] |
| AAPI | -1.617 [-6.858, 3.625] | -1.543 [-6.792, 3.705] | -1.500 [-6.742, 3.741] | -1.620 [-6.864, 3.624] |
| Other/Unknown/AIAN | 0.935 [-13.258, 15.128] | 0.852 [-13.290, 14.994] | 0.470 [-12.337, 13.277] | 0.803 [-10.602, 12.208] |
| **Education** |  |  |  |  |
| College graduate or more | -0.221 [-2.600, 2.157] | -0.266 [-2.651, 2.119] | 0.115 [-2.183, 2.414] | 0.327 [-1.981, 2.636] |
| **Time Fixed Effect** |  |  |  |  |
| Wave 2 |  |  | -0.145 [-1.001, 0.711] | -0.187 [-1.047, 0.673] |
| Wave 3 | -0.302 [-1.164, 0.560] | -0.294 [-1.156, 0.568] | -0.423 [-1.350, 0.505] | -0.455 [-1.386, 0.477] |
| Wave 4 | -0.359 [-1.254, 0.537] | -0.339 [-1.235, 0.556] | -0.420 [-1.397, 0.557] | -0.393 [-1.362, 0.577] |
| (Intercept) | -0.653 [-3.255, 1.949] | -0.717 [-3.335, 1.900] | -0.834 [-3.323, 1.655] | -0.905 [-3.502, 1.693] |
| N | 3,726 | 3,726 | 5,001 | 5,031 |
| R-Squared | 0.009 | 0.009 | 0.007 | 0.007 |
| Adj. R-Squared | 0.006 | 0.006 | 0.005 | 0.004 |
| AIC | 32306.5 | 32308.0 | 43,386.6 | 43670.7 |
| BIC | 32387.4 | 32395.2 | 43,484.4 | 43775.1 |
| RMSE | 18.39 | 18.39 | 18.45 | 18.49 |
| F-Test | F(11, 1,280) = 1.686 | F(12, 1,280) = 1.579 | F(13, 1,280) = 1.274 | F(14, 1,290) = 1.244 |

*P*-value thresholds are **P*<.05, ***P<*.01 and ****P<*.0.001

Ordinary least squares regression model predicting change in perceived breast cancer risk from perceived COVID-19 risk. Columns 1 and 2 include the restricted sample of women (N=3726) who completed two adjacent survey waves. Column 1 includes age, race, education, wave as a fixed effect and perceived COVID-19 risk from the prior wave . Column 2 adds the change in general anxiety between the prior wave and baseline. Column 3 adds to the column 1 model the change in general anxiety between the current wave and baseline. Column 4 adds to the column 1 model missed cancer screening in the prior 2 months.

AIAN=American Indian or Alaskan Native. AAPI=Asian, Native Hawaiian or Other Pacific Islander.

Reference categories: Age 40-64, Education High school or less, Race White, Perceived COVID-19 risk Very low, Time Wave 1.
